# Sintilimab-associated hemophagocytic lymphohistiocytosis: a case report

**DOI:** 10.3389/fimmu.2026.1821839

**Published:** 2026-05-20

**Authors:** Jie Zhi, Xin Wang, Bo Feng, Wujie Zhao, Bin Wang, Yitao Jia

**Affiliations:** Department of Oncology, Hebei General Hospital, Shijiazhuang, China

**Keywords:** case report, checkpoint inhibitors, esophageal squamous cell carcinoma, hemophagocytic lymphohistiocytosis, multidisciplinary discussion

## Abstract

Hemophagocytic lymphohistiocytosis (HLH) is a rare hematologic syndrome characterized by massive, uncontrolled cytokine release, which can lead to multiple organ failure and is associated with a high mortality rate. Recent studies have found that checkpoint inhibitors (ICIs) can induce HLH. This case report describes a 75-year-old man with metastatic esophageal squamous cell carcinoma (ESCC) who developed sintilimab-associated HLH, presenting as refractory fever and respiratory symptoms initially misdiagnosed as infection. The diagnosis, confirmed via elevated ferritin, sCD25, and reduced NK-cell activity, was delayed due to nonspecific symptoms. Following the initiation of dexamethasone, the patient’s symptoms resolved and laboratory parameters normalized, leading to complete remission. The case highlights HLH as a rare but life-threatening immune-related adverse event of checkpoint inhibitors, underscores the importance of early multidisciplinary discussion (MDT) in patients with unexplained fever, and demonstrates the early diagnosis and treatment are critical.

## 1 Introduction

Checkpoint inhibitors (ICIs) such as sintilimab have become a cornerstone in the treatment of esophageal cancer (EC) (1). In recent years, increasing attention has been paid to immune-related adverse events (irAEs) associated with ICIs. Among these, haemophagocytic lymphohistiocytosis (HLH) is a rare but clinically critical complication, with an incidence of less than 0.1% (2). Current evidence suggests that its pathophysiology may involve cytotoxic defects mediated by T lymphocytes and NK cells, triggering a massive release of cytokines and promoting haemophagocytic activity by macrophages (3).

HLH is divided into two main categories: primary and secondary. Secondary HLH can stem from viral infections, autoimmune diseases, malignancies, or iatrogenic factors (4). Clinical data reveals that the mortality rate of HLH is as high as 41%, and the prognosis is often worse in patients with concurrent malignancies. The primary reasons for the poor prognosis are delayed diagnosis and treatment (5). However, the clinical symptoms of HLH lack specificity and are easily confused with other diseases. Clinicians often have insufficient knowledge of this disease, which can easily lead to misdiagnosis and missed diagnosis.

This article reports a case of HLH secondary to sintilimab treatment in a patient with metastatic esophageal squamous cell carcinoma (ESCC). Initially, the patient presented with fever and respiratory symptoms, which were mistaken for an infectious etiology, leading to antibiotic treatment for over 20 days without clinical improvement. The diagnosis was subsequently confirmed through multidisciplinary discussion (MDT). Following the initiation of glucocorticoid therapy, clinical symptoms, including fever, resolved rapidly, and laboratory parameters showed significant improvement, ultimately achieving complete remission.

## 2 Case report

The patient was a 75-year-old male with an Eastern Cooperative Oncology Group (ECOG) performance status score of 1. In April 2021, he was admitted to our hospital with a chief complaint of progressive dysphagia, which had persisted for 4 months and worsened significantly over the previous month. Diagnostic evaluations, including gastroscopy, imaging studies, and pathological biopsy, confirmed a diagnosis of lower esophageal squamous cell carcinoma (cT3N1M0). He had no significant past medical history, including chronic conditions such as hypertension, coronary heart disease, or diabetes. There was also no reported family history of hereditary disorders or allergies. Following preoperative assessment, the patient underwent thoracoscopic esophagectomy with esophagogastric anastomosis and jejunostomy on May 19, 2021. Postoperative pathological report revealed poorly differentiated squamous cell carcinoma infiltrating the adventitia, with evidence of vascular tumor thrombi and perineural invasion. Both proximal and distal surgical margins were negative. Lymph node metastasis was present in the carina (1/8), but absent in the paraesophageal (0/5), left gastric (0/9), left recurrent laryngeal (0/1), and right recurrent laryngeal (0/2) lymph nodes. Immunohistochemical results: AE1/AE3 (+), CK5/6 (+), P63 (+), CK7 (–), CEA (focally +), CDX2 (–), Syn (focally +), CD56 (–), Ki67 (positive cell count 60%), poorly differentiated squamous cell carcinoma. The final pathological staging was pT3N1M0, stage IIIB. From July 5 to August 6, 2021, the patient received adjuvant radiotherapy (50 Gy in 25 fractions) following surgery. On December 24, 2024, PET/CT imaging revealed a heterogeneous, hypermetabolic soft-tissue mass at the right supraclavicular region, suggestive of metastatic esophageal carcinoma. Subsequently, from January 8 to February 17, 2025, the patient underwent radiotherapy targeting the right supraclavicular mass (50 Gy in 25 fractions). The treatment was completed uneventfully, with no reported radiation-related adverse effects. However, follow-up assessment showed no significant reduction in the size of the metastatic lesion.

On March 18, 2025, the patient initiated systemic therapy with sintilimab (200mg d1, Q21d) combined with albumin-bound paclitaxel (350mg d1, Q21d). Following the first cycle, the patient developed frequent premature atrial contractions; cardiotoxicity potentially associated with immunotherapy (grade 1) could not be excluded. Sintilimab was therefore discontinued, and albumin-bound paclitaxel was continued as monotherapy for two additional cycles. On June 3, 2025, reassessment indicated that the patient’s cardiac risk was controllable. After obtaining full informed consent, sintilimab was reintroduced in combination with albumin-bound paclitaxel for one further cycle on June 6, 2025. Subsequent efficacy evaluation indicated stable disease.

**Figure 1:**
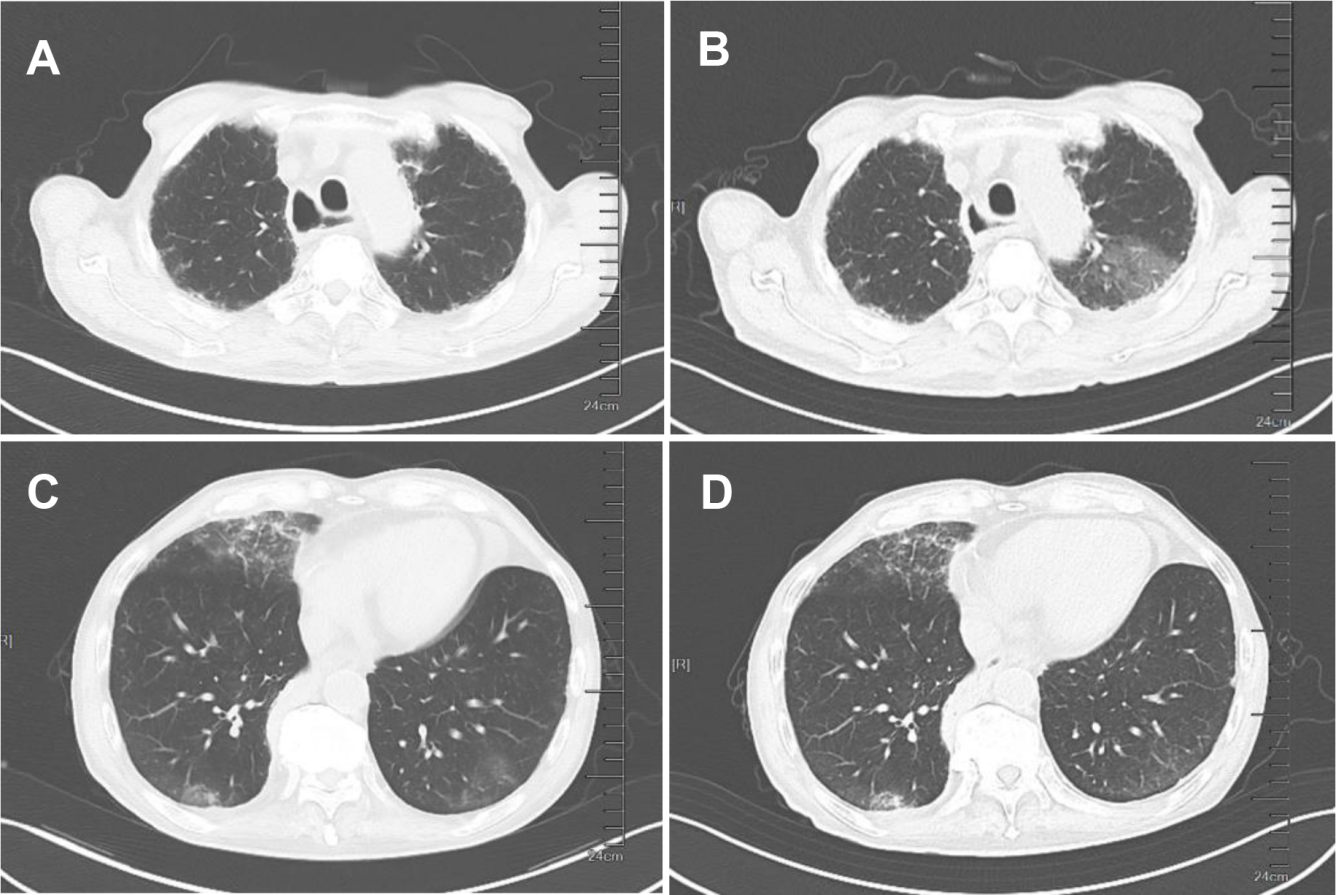
Chest CT images of the patient before and after treatment. **(A, C)** The patient’s chest CT upon admission on Day 9. **(B, D)** The patient’s chest CT after anti-inflammatory treatment on Day 14.

**Figure 2:**
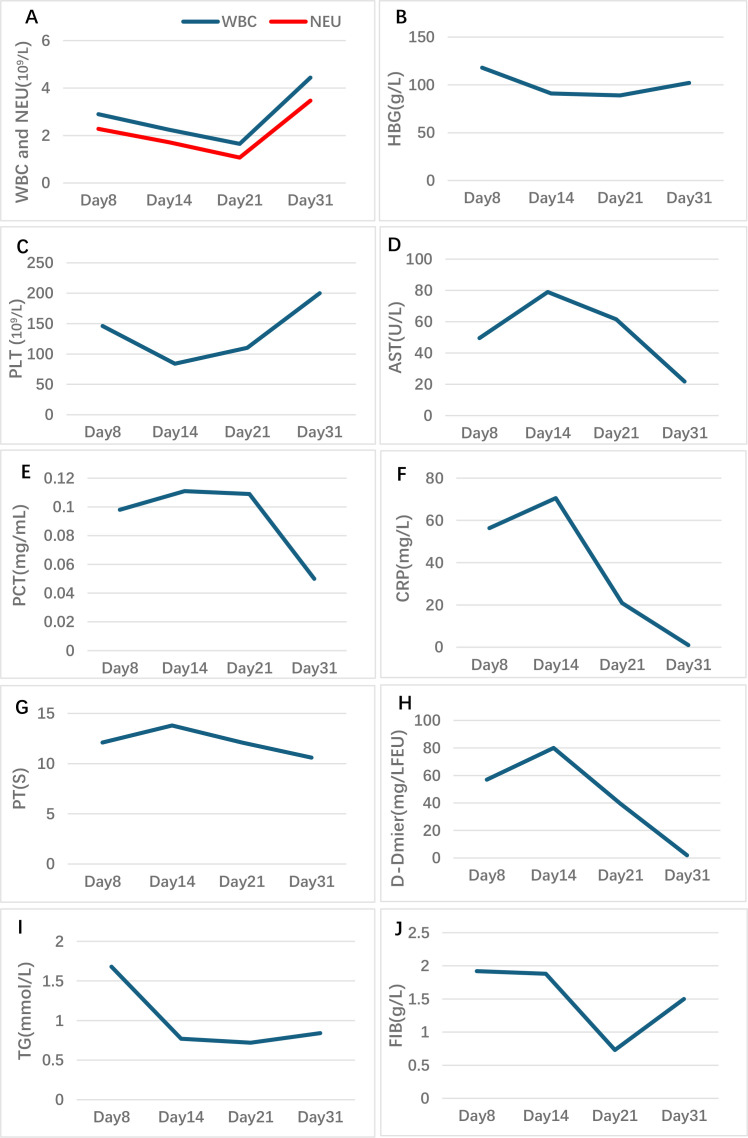
Changes in patient laboratory parameters. **(A–C)** Changes in WBC, NEU, HBG, and PLT in whole blood cell. **(D)** Changes in AST in liver function test. **(E, F)** Changes in inflammatory markers PCT and CRP. **(G, H)** Changes in PT and D-dimer in coagulation function. **(I, J)** Changes in TG and FIB.

**Figure 3:**
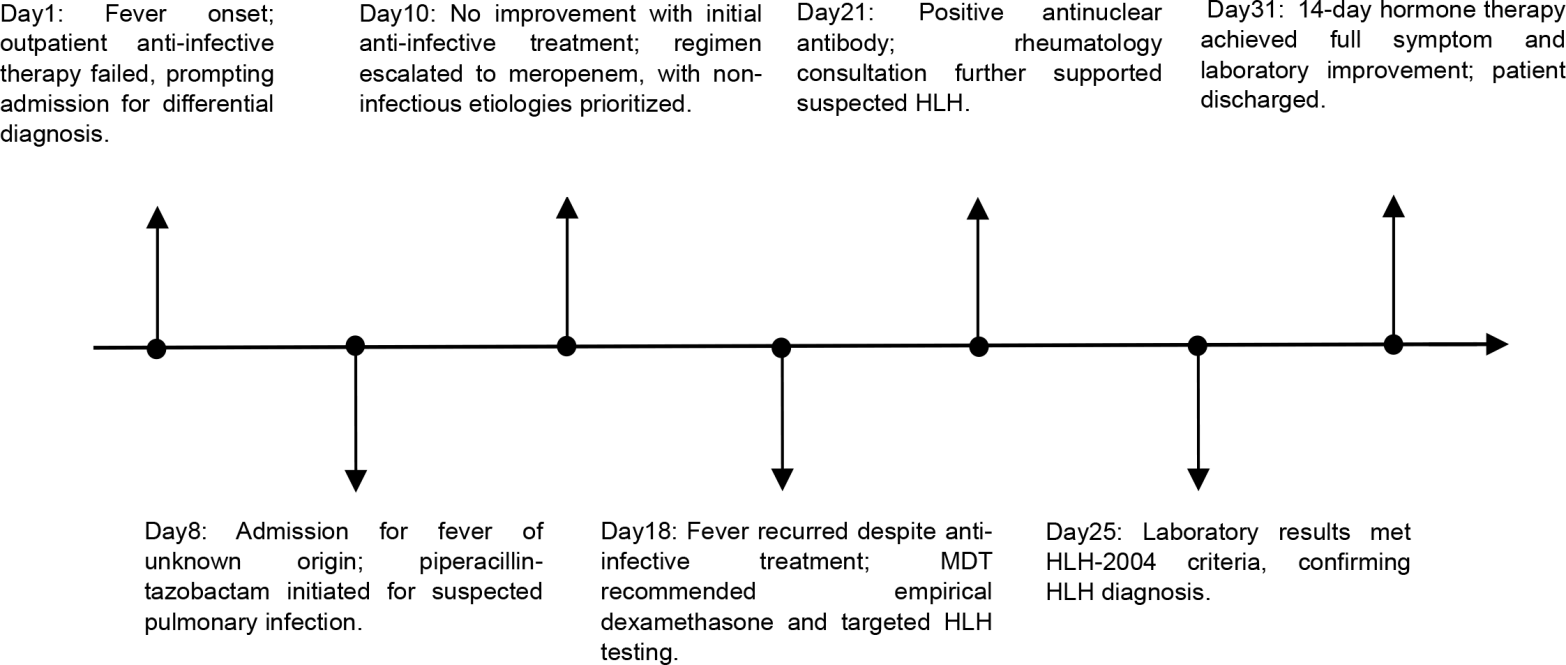
Timeline of diagnosis and treatment for the patient with patient with confirmed HLH.

On July 15, 2025 (The baseline date was defined as Day 1), the patient developed an unexplained fever, with a maximum body temperature of 38.8°C, accompanied by cough and production of white, viscous sputum, but without chills or rigors. The patient received anti−infective treatment with ceftriaxone and azithromycin at a local hospital, with poor response. On Day 8, the patient was transferred to our hospital, still presenting with intermittent high fever (up to 40°C) accompanied by chills and rigors. Body temperature could be reduced to normal with antipyretic medication, but fever recurred every 6–8 hours. On admission, the patient presented with persistent high fever; complete physical examination showed no jaundice, superficial lymphadenopathy, hepatomegaly or splenomegaly, and no other HLH-related positive signs. Chest CT revealed bilateral pulmonary interstitial fibrosis with a high likelihood of interstitial inflammation, as well as bilateral lower lobe pneumonia (**Figures 1A, C**). Laboratory findings were as follows: complete blood count: WBC:2.90×10^9/L; NEU:2.28×10^9/L; L:0.56×10^9/L; HBG: 118.00g/L; PLT: 146.00×10^9/L; liver function test: AST: 49.5U/L; CRP: 56.35mg/L; PCT: 0.098ng/mL; FER: 21440.096ng/mL; coagulation function: D-dimer: 57.02mg/L FEU; TG:1.68mmol/L; FIB:1.92 g/L; the remaining parameters were within normal limits (**Figure 2**). Tests for influenza A/B virus, respiratory syncytial virus, Aspergillus antigen, cytomegalovirus DNA, and blood cultures were all negative. Epstein−Barr virus DNA was positive at 2.88 × 10³ copies/mL. Given persistent high fever and respiratory symptoms, empirical therapy with piperacillin−tazobactam was started on Day 8. Ganciclovir was added in response to the positive EBV DNA, and omadacycline was introduced due to possible community−acquired pneumonia. Despite these interventions, fever recurred, prompting a switch to meropenem on Day 10 (**Figure 3**). Temperature control remained suboptimal. Repeat chest CT indicated bilateral pulmonary interstitial fibrosis and interstitial inflammation, with progression in the left lung and slight improvement on the right (**Figures 1B, D**).

On Day 14, repeat complete blood count showed: WBC: 2.24×10^9/L; NEU: 1.72×10^9/L; L: 0.45×10^9/L; HBG: 91.00g/L; PLT: 84.00×10^9/L; liver function test: AST: 79U/L; CRP: 70.54mg/L; PCT: 0.111ng/mL; PT: 13.8s; APTT: 45.1s; FIB: 1.83g/L; coagulation function: D-dimer: 80mg/L FEU; TG:0.77mmol/L; FIB:1.88 g/L (**Figure 2**). Follow−up chest imaging revealed mixed progression and regression of pulmonary inflammation (**Figures 1B, D**). Bronchoscopy with bronchoalveolar lavage (BAL) was performed for further evaluation. Endoscopic examination showed a small amount of secretions and an irregular airway mucosa. Cytological analysis of the BAL fluid demonstrated the following distribution among 100 non−epithelial cells: 82% neutrophils, 10% lymphocytes, and 8% mononuclear macrophages. Next−generation sequencing (NGS) of the BAL fluid detected human herpesvirus 3, human herpesvirus 4, and parvovirus B19, all with concentrations below 10³ copies/mL. Concentrated acid−fast bacilli smear, bacterial culture, and fungal culture were negative.

Following MDT involving the departments of Radiology, Pharmacy, Intensive Care, and Respiratory Medicine on Day 18, the following recommendations were made: ganciclovir was discontinued, while meropenem was continued for anti-infective therapy. In light of thrombocytopenia and coagulopathy potentially associated with omadacycline, this agent was also discontinued, and plasma transfusion was administered to correct coagulation parameters. Given the patient’s immune dysfunction, the presence of a severe infection triggered by an immunocompromised state, possibly accompanied by secondary hyperfibrinolysis, could not be ruled out. To further clarify the etiology, antinuclear antibody and vasculitis screening were recommended. In addition, low−dose steroid pulse therapy with dexamethasone (10mg iv qd) and human immunoglobulin were initiated (**Figure 3**). Following this intervention, the patient became afebrile, and meropenem was discontinued after a cumulative treatment duration of 12 days.

Subsequently, the laboratory report was received, which demonstrated a positive result on the antinuclear antibody (ANA) immunoassay. The ANA profile revealed elevated anti-SSA antibody (26.8 AU/mL) and anti-Ro-52 antibody (51.02 AU/mL). Given these immunological abnormalities, rheumatology consultation was obtained. HLH was strongly suspected, and further workup including soluble CD25 (sCD25) testing, NK-cell activity, and bone marrow aspiration was recommended. Considering the patient’s advanced age and poor general condition, bone marrow aspiration was temporarily deferred. Results returned as follows: sCD25–2055 U/mL, NK-cell activity 10.19%. In the context of rapidly progressive clinical deterioration, HLH triggered by ICIs was considered highly likely. Diagnosis of HLH was established according to the HLH-2004 criteria of the Histiocyte Society. The patient fulfilled 5 out of 8 clinical–laboratory criteria, meeting the threshold for diagnosis. A detailed summary is presented in **Table 1**.

**Table 1:** HLH-2004 diagnostic criteria and fulfillment in the present case.

2004 diagnostic criteria for secondary HLH	Patient’s findings	Fulfilled
Fever: ≥38.5°C, persistent	Sustained fever up to 40°C, refractory to antibiotics	Yes
Splenomegaly	No splenomegaly	No
Cytopenia with at least 2 of the following: HBG <90 g/L PLT <100×10^9^/L NEU <1.0×10^9^/L	PLT nadir: 84×10^9^/L; HBG nadir: 89 g/L	Yes
Hypertriglyceridemia or hypofibrinogenemia: TG ≥3.0 mmol/L or FIB ≤1.5 g/L	FIB nadir:0.72 g/L	Yes
Hemophagocytosis in bone marrow or spleen or lymph nodes or liver	Bone marrow aspiration not performed	No
Ferritin ≥500 ng/mL	21440.096 ng/mL	Yes
Low or absent NK-cell activity	10.19% (normal: 15.11%–23.70%)	Yes
Soluble CD25 (sIL-2R) ≥2400 U/mL	2055 U/mL ((normal:223–710 U/mL)	No

Treatment with intravenous dexamethasone (10mg iv qd) was continued for a full 2-week course, followed by transition to oral dexamethasone (5mg qd) with tapering by half every 2 weeks until reaching 1.25 mg, which was maintained for an additional 2 weeks before discontinuation. Total corticosteroid duration was planned for 8 weeks to prevent relapse from overly rapid tapering. Throughout this period, oral voriconazole and sulfamethoxazole were administered for prophylaxis against fungal infection (**Figure 3**).

Fourteen days after initiating corticosteroid therapy, laboratory results were as follows: WBC: 4.44×10^9/L; NEU: 3.47×10^9/L; L: 0.79×10^9/L; HBG: 102.00g/L; PLT: 200.00×10^9/L; CRP: 1.01mg/L; PCT: 0.04ng/mL; FER: 3900ng/mL; coagulation function showed a D-dimer level of 1.98mg/L FEU; TG:0.84mmol/L; FIB:1.5 g/L; and liver function within normal limits (**Figure 2**). The patient remained afebrile with improved overall condition and was subsequently discharged (**Figure 3**). No recurrence of HLH−related symptoms was noted during follow−up.

## 3 Discussion

This report describes a case of HLH associated with cancer immunotherapy. HLH is a life-threatening systemic hyperinflammatory syndrome driven by impaired cytotoxic function of T, NK cells and macrophages, resulting in an uncontrolled cytokine storm (IFN-γ, IL-1, IL-6, IL-18, TNF-α), diffuse tissue injury and multiorgan failure (6, 7). Without timely intervention, HLH has an extremely poor prognosis, with a median survival of less than two months (8). The patient initially presented with refractory fever and respiratory symptoms misdiagnosed as pulmonary infection, with no response to over 20 days of broad-spectrum anti-infective therapy. The diagnosis of HLH was confirmed via MDT assessment, with fulfillment of 5 out of 8 HLH-2004 diagnostic criteria (fever, bicytopenia, reduced NK cell activity, markedly elevated ferritin, low FIB level), meeting the global gold standard for HLH diagnosis. The patient achieved rapid and complete remission after first-line corticosteroid monotherapy, with no recurrence during follow-up.

HLH diagnosis was primarily based on the 2004 Histiocyte Society diagnostic criteria, integrating clinical and laboratory findings (9). Primary HLH is mostly genetic and prevalent in children, while secondary HLH, more common in adults, is typically triggered by infection, malignancy, or autoimmune disease; lymphoma accounts for up to 76% of malignancy-associated cases (10). We systematically excluded alternative etiologies of secondary HLH in this case: malignancy-associated HLH was ruled out as the patient’s metastatic ESCC remained stable with no tumor progression or lymphoma; autoimmune disease-associated HLH was excluded via rheumatology consultation, with positive autoantibodies attributed to ICI-mediated immune activation rather than primary connective tissue disease; infection-associated HLH (especially EBV-related) was excluded given non-response to full-course anti-infective/antiviral therapy, negative comprehensive pathogenic testing, and low-level EBV viremia insufficient to drive HLH. The patient’s last dose of sintilimab was administered on June 6, 2025, and the first unexplained fever occurred on July 15, 2025, with an interval of 5 weeks. This interval fully falls within the well-documented typical onset time window of ICI-related HLH: published studies consistently show that the median onset time of ICI-associated HLH is 4–12 weeks after the first ICI dose, with delayed onset up to 6 months after the last dose. Combined with the clear temporal correlation between symptom onset and sintilimab reintroduction, and rapid complete response to glucocorticoid therapy targeting ICI-induced immune hyperactivation, sintilimab-mediated uncontrolled immune activation was confirmed as the primary HLH trigger. Consistent with adult HLH clinical consensus, a synergistic effect of low-level EBV viremia on disease progression cannot be fully excluded. We also performed a formal quantitative causality assessment between sintilimab exposure and HLH using the internationally validated Naranjo Adverse Drug Reaction Probability Scale. The total score of this case was 7 points, corresponding to a Probable adverse drug reaction. This further validates our results (**Supplementary Table 1**).

In this case, the ESCC patient initially presented with fever and respiratory symptoms, with only mild leukocyte abnormalities on early complete blood count that failed to raise timely vigilance. The chest CT showed bilateral interstitial inflammation. Both sintilimab-induced immune pneumonitis and HLH-related pulmonary damage share the same core pathogenesis: PD-1 inhibitor-mediated excessive immune activation. We therefore interpret the bilateral interstitial inflammation as a combined result of both concurrent sintilimab-induced immune-mediated pneumonitis and HLH-related systemic pulmonary involvement, which also explains the patient’s excellent response to glucocorticoid monotherapy (11–13). Subsequent bronchoscopy identified no clear infectious source, and empirical antimicrobial therapy was ineffective, preliminarily ruling out infection. With disease progression, the patient developed progressive cytopenia, markedly elevated ferritin, decreased NK-cell activity, and elevated sCD25, leading to the final HLH diagnosis. The main reasons for the delayed diagnosis in this case were: first, insufficient clinical awareness—nonspecific symptoms such as fever and cough in cancer patients are often attributed to infection or the underlying malignancy, with inadequate vigilance toward rare but critical immunotherapy−induced complications like HLH; second, atypical early presentation—the patient initially exhibited only mild hematologic changes, while hallmark features such as significant cytopenia and hyperferritinemia became evident only as the disease progressed. To reduce misdiagnosis, we recommend leveraging MDT and proactively involving rheumatology−immunology specialists in the evaluation of cancer patients receiving immunotherapy who present with unexplained fever.

Currently, there is no standardized treatment protocol for ICIs-induced HLH. In the present case, glucocorticoid therapy effectively controlled the patient’s fever, with gradual improvement in complete blood counts, coagulation parameters, and liver function. After two weeks, treatment was transitioned to oral dexamethasone with a slow tapering schedule. Subsequently, the patient’s hematologic parameters normalized, and liver function, serum ferritin, and coagulation profiles showed significant improvement, accompanied by marked clinical recovery. Our patient’s HLH treatment regimen was developed via multidisciplinary assessment in line with international HLH and ICI toxicity guidelines. The etoposide-based HLH regimen is the gold standard for high-risk adult HLH per the 2019 ASH consensus, while NCCN and ESMO guidelines recommend corticosteroid monotherapy as first-line treatment for non-life-threatening ICI-associated HLH, supported by its distinct immune-driven pathogenesis and the proven efficacy of corticosteroids in suppressing cytokine storm (14, 15). In this case, corticosteroid monotherapy was selected as first-line treatment in accordance with international guidelines for immune-related HLH. Standard etoposide-containing regimens were not used because the patient was elderly, had baseline cytopenia and coagulopathy, and showed no life-threatening organ dysfunction or lymphoma.

The clinical features, diagnostic trajectory and therapeutic strategy of our case align with the well-characterized phenotypes of ICI-associated HLH reported in multiple published clinical studies. The case of ICI-associated HLH in a glioblastoma patient reported by Alan et al. shares core clinical parallels with our own: symptom onset within 6 weeks of the final ICI dose, initial misclassification as refractory infection, and high-dose corticosteroids as first-line treatment. However, the patient in that study exhibited no response to first-line corticosteroid monotherapy, requiring salvage cytokine-directed therapy with tocilizumab, and ultimately succumbed to underlying tumor progression despite partial laboratory improvement (16). By contrast, our 75-year-old patient achieved sustained complete remission with corticosteroid monotherapy alone, adding critical real-world evidence supporting the efficacy of this regimen in appropriately selected patients. Further, Guzel et al. noted that ESCC accounts for only 1.2% of all ICI-associated HLH cases, with very few documented sintilimab-related cases despite the agent’s widespread use in the first-line treatment of advanced ESCC (17). In parallel, a landmark multicenter study has established the definitive core clinical, diagnostic, and therapeutic framework for ICI-associated HLH (18). Our case fully conforms to this paradigm across all key domains: onset timing, canonical clinical and laboratory features, initial misdiagnosis of fever as infectious etiology, and adherence to evidence-based diagnostic and treatment principles. Taken together, this case validates the universality of the established clinical framework for ICI-associated HLH, and provides rare real-world data on sintilimab-induced HLH in ESCC to facilitate earlier recognition of this life-threatening immune-related adverse event.

We explicitly acknowledge the following limitations of this case report (1): bone marrow aspiration was not performed due to the patient’s advanced age, high bleeding risk, and refusal of the procedure, which limits the definitive confirmation of hemophagocytosis; (2) definitive causality cannot be established due to the absence of drug re-challenge (not performed for ethical and safety reasons); (3) this is a single case report, and the treatment efficacy needs to be validated in larger prospective cohorts.

HLH is a systemic inflammatory syndrome with a high case fatality rate and is often difficult to distinguish from infection or other complications during immunotherapy (19, 20). Clinically, multidisciplinary collaboration should be promptly initiated for suspected cases, and specific diagnostic markers should be incorporated to enable early intervention and improve prognosis.

## Data Availability

The original contributions presented in the study are included in the article/**Supplementary Material**. Further inquiries can be directed to the corresponding author.
