# Supplementary material for: Sintilimab-associated hemophagocytic lymphohistiocytosis: a case report
Source: Front Immunol. 2026 May 20;17:1821839. doi: 10.3389/fimmu.2026.1821839 (PMC13230065; doi:10.3389/fimmu.2026.1821839)
Supplement: Supplementary file 1 [file Table1.docx]

Supplemental Table 1. Naranjo Scale Causality Assessment for Sintilimab-Associated HLH

| Naranjo Scale Item | Assessment for the Present Case | Score |
| --- | --- | --- |
| Are there previous conclusive reports on this reaction? | Yes, multiple published studies have reported HLH as a rare but severe immune-related adverse event of sintilimab and other ICIs. | 1 |
| Did the adverse event appear after the suspected drug was administered? | Yes, HLH-related symptoms occurred 5 weeks after the last dose of sintilimab, with a clear sequential relationship. | 2 |
| Did the adverse event improve when the drug was discontinued and specific antagonist (glucocorticoid) was administered? | Yes, the patient’s fever resolved rapidly within 48 hours after sintilimab discontinuation and glucocorticoid initiation, with progressive normalization of all laboratory parameters. | 1 |
| Did the adverse event reappear when the drug was readministered? | Sintilimab was not readministered due to safety concerns. | 0 |
| Are there alternative causes that could on their own have caused the adverse event? | No, we have systematically ruled out infection (including EBV-related HLH), malignancy progression, and primary autoimmune disease as independent triggers of HLH. | 2 |
| Did the adverse event reappear when a placebo was given? | Not applicable. | 0 |
| Was the drug detected in blood/body fluids at concentrations known to be toxic? | Not applicable for monoclonal antibody biologics with no defined toxic blood concentration. | 0 |
| Was the adverse event more severe with increased dose, or less severe with decreased dose? | Not applicable, fixed standard dose of sintilimab was used. | 0 |
| Did the patient have a similar reaction to the same or similar drugs in previous exposure? | No prior exposure to PD-1 inhibitors other than sintilimab. | 0 |
| Was the adverse event confirmed by objective evidence? | Yes, the diagnosis was confirmed by objective laboratory markers (ferritin, sCD25, NK cell activity) and clinical treatment response. | 1 |
| Total Score |  | 7 |
